# On the Puncture of the Urinary Bladder

**Published:** 1805-01-01

**Authors:** T. L. O. Davies


### On the Puncture of the Urinary Bladder.

### by Mr. T. L. O. Davies.

I Was requested, on Sunday the 17th of June last, by a brother practitioner, to visit John Morley, a boy of about eleven years of age, who on the preceding Thursday had received a very considerable contusion on the perinæum, owing to his falling across some pales he was endeavouring to walk on.

The boy had passed no urine since the accident happened, and he was in the greatest agony, suffering from the over distension of the bladder. The perinæum, scrotum, and parts about, were exceedingly tense and swelled, and the cellular membrane so much loaded with extravasated blood, as to resemble in appearance, gangrene. Every thing that art could suggest had, I believe, been tried by the gentleman that first attended him. He had been been bled, and his bowels thoroughly evacuated. Fomentations had been applied, and the warm bath also repeatedly used. The catheter had been several times attempted, but neither that nor the smallest bougie could be introduced. The case proving thus obstinate, (the pain increasing rapidly, indeed it was now excruciating, not a drop of urine having passed for at least seventy-two hours, and the bladder so amazingly tense and swelled, and extended nearly up to the scrobiculus cordis,) it appeared to me, no time was to be lost to endeavour to save the life of the child. I therefore recommended the puncture of the bladder immediately, but in what part became a matter of momentary deliberation. Surgeons are still divided in opinion, I believe, which is the safest and most advantageous way of getting into the bladder. In this case, the inflammation and tension of the parts absolutely forbid the puncture through the perinæum; and though the puncture through the rectum may possibly be, by the greater number, considered as the most eligible way, yet here I strenuously recommended the puncture above the pubis, as being more easily and readily performed, and less likely from its distance to the injured part, to increase the inflammation already formed.

Accordingly, a trocar, with a canula exactly two inches in length, was plunged into the bladder, nearly or about one inch and a half above the pubis, and two quarts or more of urine were immediately drawn off. The child experienced immediate relief; he was put into bed; the canula was suffered to remain in, having a cork fitted to it; and some holes having previously been drilled in the upper part, or shoulder, it was kept steady in its place by some tapes affixed to it, and fastened round the body, having a compress of linen and a T bandage over the whole. The child slept well the following night; in the morning the cork was taken out, and the water drawn off. By attention to regimen, and the child's bowels, no great degree of fever ensued, neither any tension or inflammation about the punctured part. The fomentation was continued to the perinæum and scrotum, and those parts were constantly covered with a poultice of bread and milk, by which means the extravasated blood in the cellular membrane became gradually absorbed. Not being in the habit of attending the patient regularly myself, I could not accurately note every symptom that occurred; but about the seventh day from the accident, we felt a fluctuation of matter in the perinæum, and I understood the boy boy had had more pain there for the last two or three days. A puncture with the lancet was therefore immediately made, and a large quantity of ichorous matter let out. The wound was superficially dressed. The urine had been, and was still, daily twice or thrice drawn off through the canula, and a bougie also attempted to be introduced. About the twelfth day from the accident, we discovered that the boy, when endeavouring to make water, forced some urine through the wound in the perinæum, and we shortly after saw that a small quantity passed the natural passage. A small bougie was now introduced into the bladder, and suffered to remain there some hours. I wished, considering the canula had been in twelve days, without being once removed, now to take it out; but my friend not agreeing with me in that opinion, I consented to its continuance in the bladder two days longer. It was taken out then, and with great ease. The wound made by the trocar healed very readily, and the urine was evacuated through the urethra, and also through the wound in perinæo. The boy's health was tolerably good, except being rather reduced, we therefore gave him the cinchona three or four times a day; and in the course of two or three weeks, the wound in the perinæum was completely healed also. The abdomen became somewhat enlarged after the canula had been removed; it had a dropsical appearance, some urine, no doubt, escaped into its cavity; but by the use of a flannel bandage, and embrocating the part with a strong camphire liniment, the size of the belly diminished daily, and in about five weeks from the accident, the boy was in every respect cured.

To draw any inference from the successful termination of one solitary case, would be both unfair and futile. It is probable that in every case, where it is necessary to puncture the bladder, each mode, according to particular circumstances, may in its turn have a decided preference; but if I may venture an opinion, I should conceive there can scarcely ever be a well founded objection to the puncture above the pubis. The arguments I have only heard against it are, 1st. The necessity of leaving in a canula for so great a length of time, as may be necessary before the obstruction in the urethra is removed, and the difficulty and pain occasioned thereby in extracting it. 2dly. The puncture not being made in a depending part. In this case, the first of these objections was entirely done away; the canula, though it had been in the bladder fourteen teen days, without being either by design or accident once removed, was taken out with the greatest ease. In regard to the second objection, the bladder was not only nearly emptied every time the cork was taken out of the canula, but a considerable quantity of an increased discharge of mucus was evacuated through the canula also, for three or four days before it was extracted.

### On the Bronchocele.

A lady, between forty and fifty years of age, applied to me about the beginning of the month of November, 1802, on account of a large tumour she had on the fore part of her neck. It had been, she told me, ten years growing, and was now increased to such a size, that she was obliged by her dress to hide the deformity. She had some time before applied to a surgeon, who had tried some external stimulants and mercurials, but in vain; and indeed, considering her time of life, I did not myself conceive that any medicine could be of use to her. Recollecting, however, in some former numbers of the Medical and Physical Journal, cases in which the efficacy of burnt sponge had been very strongly marked, I recommended her to try that medicine, as the only remedy I knew of. Though the lady suffered no inconvenience from the swelling, yet its having greatly increased of late, alarmed her, and she was determined to give the sponge a fair trial. I accordingly made some lozenges, each of which contained ʒß of burnt sponge. Of these she took three daily, by laying them under the tongue till they gradually dissolved; and in this way, without any interruption, she continued taking the lozenges, until she had swallowed sixteen ounces of the sponge. Not the least nausea was ever excited by it; but at the expiration of that time, no diminution whatever of the tumour taking place, my patient began to be disgusted, and was rather averse to continue the medicine longer; nor could I in my conscience request her. Her health was good, and she made her mind up to the deformity, on my assuring her, that such diseases were very rarely of any serious consequence.

The beginning of September, in the following year, I was sent for by the lady, on account of her health; she had lost her appetite, was grown thin, was feverish, &c. I looked at the tumour; it appeared of nearly the same size as when I saw it the year before. To mention more particularly her complaints is here unnecessary; but apprending prehending some chronic inflammation about the liver, I thought it right, after giving some purgative medicines, to put her on a plan of deobstruents, with small doses of calomel. On this plan she continued till the beginning of November, when her health was perfectly re-established. At this time the tumour remained exactly in the same state: but I was most exceedingly surprized in about a week; after the lady called on me to show her neck, when it was diminished more than one half, and in a few days more, was wholly gone. The total dispersion of it was the work only of about ten days, and there is not at this moment any prospect of its re-appearance. Here now is a case, in which the sponge had no efficacy, and yet a curable case. To what cause then can we attribute the resolution of the tumour? Was it owing to the continued use of calomel? or, was it merely the effect of chance?

The true cause and nature of this disease is ill understood; that they are not all similar, I think beyond doubt; and that the contents of the bronchocele in my patient was water, I think as clear. Most probably the water was contained in a cyst or cysts, which bursting, occasioned the tumour so quickly to disappear; and I think I recollect hearing that the late Dr. Hunter dissected an enlarged thyroid gland, and found it made up of cysts containing water.

*Alresford, Hants, Nov.* 7, 1804.

